# Encapsulation of Cynara Cardunculus Guaiane-type Lactones in Fully Organic Nanotubes Enhances Their Phytotoxic Properties

**DOI:** 10.1021/acs.jafc.1c07806

**Published:** 2022-03-15

**Authors:** Francisco J.R. Mejías, Inmaculada P. Fernández, Carlos Rial, Rosa M. Varela, José M.G. Molinillo, José J. Calvino, Susana Trasobares, Francisco A. Macías

**Affiliations:** † Allelopathy Group, Department of Organic Chemistry, Institute of Biomolecules (INBIO), School of Science, University of Cádiz, Campus CEIA3, C/ República Saharaui, 7, Puerto Real, Cádiz 11510, Spain; ‡ Departamento de Ciencia de Los Materiales e Ingeniería Metalúrgica y Química Inorgánica, Facultad de Ciencias, Universidad de Cádiz, C/ República Saharaui, 7, Puerto Real, Cádiz 11510, Spain

**Keywords:** encapsulation, cynara cardunculus, organic nanotubes, cynaropicrin, grosheimin, aguerin B, phalaris arundinacea, lolium perenne, portulaca oleracea, STEM−XEDS, weed bioassay

## Abstract

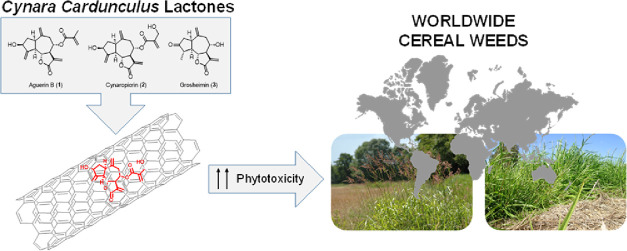

The encapsulation of bioactive natural products has emerged as a relevant tool for modifying the poor physicochemical properties often exhibited by agrochemicals. In this regard, natural guaiane-type sesquiterpene lactones isolated from *Cynara cardunculus* L. have been encapsulated in a core/shell nanotube@agrochemical system. Monitoring of the F and O signals in marked sesquiterpenes confirmed that the compound is present in the nanotube cavity. These structures were characterized using scanning transmission electron microscopy–X-ray energy-dispersive spectrometry techniques, which revealed the spatial layout relationship and confirmed encapsulation of the sesquiterpene lactone derivative. In addition, biological studies were performed with aguerin B (**1**), cynaropicrin (**2**), and grosheimin (**3**) on the inhibition of germination, roots, and shoots in weeds (*Phalaris arundinacea* L., *Lolium perenne* L., and *Portulaca oleracea* L.). Encapsulation of lactones in nanotubes gives better results than those for the nonencapsulated compounds, thereby reinforcing the application of fully organic nanotubes for the sustainable use of agrochemicals in the future.

## Introduction

**Figure 1:**
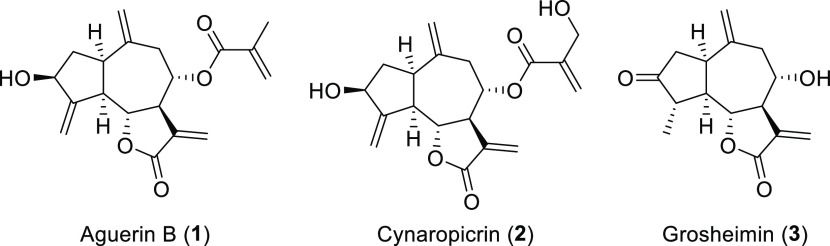
Structure of sesquiterpene lactones mainly isolated from the aerial parts of *Cynara cardunculus*.

The guaianolide-type skeleton of sesquiterpene lactones makes these substances some of the most promising phytotoxic secondary metabolites employed as natural product-based herbicides.^1−3^ The application of allelopathy is crucial for the sustainable development of agriculture, and sesquiterpene lactones, which are released by plants to defend themselves against other plants that are competing for resources, have played a key role as lead compounds in this field. *Cynara cardunculus* is a good source of these sesquiterpene lactones, with aguerin B (**1**), cynaropicrin (**2**), and grosheimin (**3**) (Figure 1) being perhaps the most abundant and active guaianolides. Indeed, their phytotoxicity against Brachiaria [*Uruchloa decumbens* (Stapf) R.D. Webster] and barnyard grass (*Echinochloa crus-galli* L.) even exceeds that for commercial herbicides such as Logran,^4^ and their use against other weeds that infect key crops, such as wheat or carrots, could solve problems related to soil persistence and pollutant phenomena that are usually associated with classical herbicides.^5−8^ In addition, the activities of *C. cardunculus* sesquiterpene lactones are boosted when they are applied together to fight weeds. As such, this joint action makes them attractive as it avoids the need for a complex isolation or purification after synthesis.^9^

The physicochemical properties and abundance of these metabolites limit their potential and widespread application as a new green herbicide. The application of low concentrations of natural products to the soil to fight weeds, in combination with large-scale production, would reduce the use of classical, nondegradable herbicides and their possible ingestion by humans as a result of their persistence in edible crops. However, properties such as bioavailability and soil stability still need to be improved to obtain the ideal natural weedkiller.

In this regard, fully organic encapsulation has become a relevant technique that has been previously applied in the agricultural field with satisfactory results. Specifically, polymeric nanoparticles comprising strigolactone mimics and inuloxin A encapsulated in cyclodextrin have been employed to fight parasitic plants.^10,11^ More recently, nanotubes generated from steroid acid were used to improve the properties of *ortho*-disulfides, which showed potent inhibitory activity against etiolated wheat coleoptiles.^12^ This latter method improves biorecognition by plant cells and offers a larger host space to increase the number of biomolecules encapsulated.

In this work, we have successfully encapsulated three of the most important guaianolides from *C. cardunculus* inside lithocholic acid nanotubes. These organic materials were characterized by transmission electron microscopy (TEM) to determine the spatial layout of the guaianolides, previously labeled with a halogen group, inside the nanotube. This procedure was found to improve the phytotoxicity of the sesquiterpene lactones, which were found to exhibit a markedly higher inhibition of germination, roots, shoots, and elongation compared with their nonencapsulated counterparts. *In vitro* experiments gave excellent results using an undifferentiated plant model (etiolated wheat coleoptile) and *Phalaris arundinacea*, *Lolium perenne,* and *Portulaca oleracea*, which infect most cereal and other relevant crops, such as wheat and carrots, with relevance all over the world, specifically in North America.^6,7,13^

## Experimental Section

### Isolation of Sesquiterpene Lactones

Stem and dry leaves from *C. cardunculus* var. *scolymus*, previously collected in Jerez de la Frontera (Cadiz, Spain), were powdered using an industrial mill and degreased with 5 L of *n*-hexane in an ultrasound bath. A total of 2.3 kg of dry material was obtained after this process. The bioactive compounds were then isolated using the method reported by Rial et al.^4^ Finally, 122.2 mg of cynaropicrin (5.24 × 10^–3^% yield), 288.6 mg of grosheimin (1.24 × 10^–2^% yield), and 90.4 mg of aguerin B (3.87 × 10^–3^% yield) were obtained (as wt % of dry plant material).

### Synthesis of 4-Fluorobenzoylaguerin B (**4**)

30 mg of aguerin B (9.09 × 10^–5^ mol) was dissolved in 2 mL of dry pyridine under an inert atmosphere. A total of 1.5 equiv of 4-fluorobenzoyl chloride (1.36 × 10^–4^ mol, 16.1 μL) was then added to the flask, and the reaction mixture was stirred for 12 h at room temperature. The mixture was subsequently extracted three times with a saturated solution of CuSO_4_, and the organic phase was washed three times with 0.1 M NaOH solution. Chromatographic separation was carried out using a gradient from 0 to 50% Hex/EtOAc. No further purification was needed. See Table S1 for NMR data. Calculated *m/z* for [C_26_H_25_O_6_F]Na^+^ 475.1533, obtained 475.1535. IR (cm^–1^): 2927.4, 1769.5, 1716.7, 1603.9, 1507.8, 1270.5, 1153.8, 1116.7, 1014.3, 964.4, 855.8, and 768.2. UV (CH_3_CN), λ_max_: 231 nm.

### Synthesis of Bis(4-Fluorobenzoyl)cynaropicrin (**5**)

30 mg of cynaropicrin (8.66 × 10^–5^ mol) was dissolved in 2 mL of dry pyridine under an inert atmosphere. A total of 3 equiv of 4-fluorobenzoyl chloride (2.60 × 10^–4^ mol, 30.7 μL) was then added to the flask, and the reaction mixture was stirred for 12 h at room temperature. The mixture was subsequently extracted three times with a saturated solution of CuSO_4_, and the organic phase was washed three times with 0.1 M NaOH solution. Chromatographic separation was carried out using a gradient from 0 to 30% Hex/acetone, and then, high-performance liquid chromatography (HPLC) was employed to isolate the product at a retention time of 7.4 min (35% Hex/acetone) (45% yield) using a Merck-Hitachi system (Tokyo, Japan) with a refractive index detector (Elite LaChrom L-2490). A semipreparative LiChrospher 10 μm 250–10 Si 60 (Merck) column was employed with a flow rate of 3 mL/min. See Table S2 for NMR data. Calculated *m/z* for [C_33_H_28_F_2_O_8_]Na^+^, 613.1644, obtained 613.1644. IR (cm^–1^): 2927.7, 1770.2, 1722.3, 1603.2, 1508.2, 1270.3, 1241.1, 1153.6, 1113.7, 854.7, and 768.1. UV (CH_3_CN), λ_max_: 241 nm.

### Synthesis of 4-Fluorobenzoylgrosheimin (**6**)

30 mg of groshiemin (1.12 × 10^–4^ mol) was dissolved in 2 mL of dry pyridine under an inert atmosphere. A total of 1.5 equiv of 4-fluorobenzoyl chloride (1.72 × 10^–4^ mol, 20.3 μL) was then added to the flask, and the reaction mixture was stirred for 12 h at room temperature. The mixture was subsequently extracted three times with a saturated solution of CuSO_4_, and the organic phase was washed three times with 0.1 M NaOH solution. Chromatographic separation was carried out using a gradient from 0 to 50% Hex/EtOAc. No further purification was needed. See Table S3 for NMR data. Calculated *m/z* for [C_22_H_21_O_5_F]Na^+^, 407.1271, obtained 407.1271. IR (cm^–1^): 2920.2, 2851.4, 1734.2, 1632.8, 1541.7, 1267.3, and 1031.7. UV (CH_3_CN), λ_max_: 234 nm.

### Synthesis of Lithocholic Acid Nanotubes

The method reported by Mejías et al. was used.^12^

### Encapsulation of Sesquiterpene Lactones inside Lithocholic Acid Nanotubes

Three sesquiterpenes, namely, aguerin B (**1**), cynaropicrin (**2**), and grosheimin (**3**), and their respective 4-fluorobenzoyl derivatives, were each encapsulated using an *in situ* method. Thus, 30 mg of the guaiane compound and 60 mg of lithocholic acid (1:2 m/m) were dissolved in 0.1 M NaOH (100 mL), and then, the mixture was stirred while adding 1.0 M HCl to neutral pH (pH ∼7.4). The solution become micellar and was stirred for a further 24 h. The sample was then dialyzed for 10 min with type-II water using a membrane with a 12,000 Da cutoff. The solution inside the membrane was recovered and stored at 4 °C for further study.

### Electron Microscopy

Samples were prepared by placing one drop of the sample dispersed in type-I water on a Lacey-carbon-coated 300 mesh copper grid. The prepared TEM grid was dried overnight on filter paper to evaporate the water before being introduced into the electron microscope.

Nanoanalytical information for the sample was obtained using an FEI Titan Cubed Themis 60–300 microscope operating at 300 kV. Double-aberration-corrected scanning TEM (STEM) was equipped with a Super X-G2 X-ray energy-dispersive spectrometer, thus providing a tool to simultaneously combine spectroscopy and image signals. The large-area views of the samples were recorded using the scanning high-angle annular dark field detector. X-ray energy-dispersive spectrometry (XEDS) analysis was performed acquiring collections of 1300 × 1430 nm size XEDS maps of the C (0.277 keV) and F (0.675 keV) signals. To improve visualization, the elemental maps were postfiltered using an Average 3, as provided for in Velox software.

### NMR Studies

NMR spectra were recorded using an Agilent INOVA-500 spectrometer equipped with a 5 mm ^1^H–^13^C–^15^N cryoprobe. The ^1^H (499.772) and ^13^C (125.826) NMR spectra were recorded in chloroform-d1 (Merck, Darmstadt, Germany) at room temperature. The concentration of each compound was 1 mg/mL. The chemical shifts are given on the δ scale and are referred to the residual chloroform (δH 7.26 and δC 77.0 ppm).

### Quantification of Encapsulated Compounds

Sesquiterpene lactones and fluorine derivatives were quantified by ultra-HPLC (UHPLC)–mass spectrometry (MS) employing a Brucker EVOQ model with triple quadrupole mass spectrometer. A Phenomenex Kinetex 1.7 μm C18 column (100 × 2.1 mm and a particle size of 1.7 μm) was employed, along with an APCI ionization source. The method employed was a modification of multiple reaction monitoring (MRM) published in the literature to include the fluorinated compound 4-fluorobenzoylgrosheimin.^14^ The dependent parameters for each compound and the internal standard employed (santamarine) were optimized by direct injection into the mass spectrometer to get a maximum MRM signal intensity for the ammonium adduct [M + NH_4_]^+^. Table 1 shows the precursor ions and subsequent fragments obtained by MRM analysis, as well as the collision energy required to achieve these fragmentations.

**Table 1:** Mass-Charge Ratio and Specific Parameters for Products and Internal Standards (I.S)

		quantifier ion (*m/z*)			qualifier ion (*m/z*)		
compounds	*t*_R_ (min)	precursor ion (Q1)	product ion (Q3)	E.C (eV)	precursor ion (Q1)	product ion (Q3)	E.C (eV)
grosheimin	8.36	280	263	3	281	149	10
cynaropicrin	9.40	364	227	10	364	245	5
aguerin B	9.87	348	227	9	348	245	6
4-fluorobenzoylgrosheimin	10.23	402	385	6	402	245	10
santamarine (I.S)	9.67	266	231	6	266	185	10

Each compound was dissolved in MeOH to get a calibration curve with concentrations from 50 to 0.1 mg/mL. Santamarine was employed as the internal standard due to its similar structure and molecular weight and the fact that it is a sesquiterpene lactone from the Asteraceae family. Furthermore, this compound is not produced by *C. cardunculus*. The calibration curve was obtained as a ratio between the compound peak area and the internal standard peak area. This curve was fitted to a lineal function weighted by 1/*nx* (*R*^2^ > 0.99), where “*n*” is the number of calibration standards.

Sesquiterpene lactones and their derivatives were determined using the MRM method by comparing the retention times in the chromatograms, and the most stable transitions were calculated upon direct injection into the mass spectrometer.

### Encapsulation Percentage Calculation

The percentage of each sesquiterpene lactone inside the nanotubes was determined by disrupting the supramolecular structure. To release the molecules, samples were dissolved in methanol and shaken using a vortex mixer. The solution was then filtered through a 0.22 μm polytetrafluoroethylene (PTFE) filter, and this new sample was mixed with 5 μL of the internal standard (1 mg/mL) before being injected into the UHPLC column.

### Solubility Enhancement Calculation

To calculate the improvement in water solubility for the sesquiterpene lactones and derivatives, a 1.0 mg/mL solution was prepared in water. These samples were then filtered through a PTFE 0.22 μm filter, and their areas in UHPLC were recorded for comparison with the previous calibration curve measured for MeOH (similar molar extinction coefficient for water and MeOH).^15^ This value (maximum water solubility of the compound) was compared with the encapsulated compound using the formula
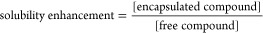


### Etiolated Wheat Coleoptile Bioassay

All experiments were carried out following the procedure reported in the literature, with some minor modifications.^11,12^ Sesquiterpene lactones and their derivatives encapsulated inside lithocholic acid nanotubes were added diluted in an aqueous phosphate-buffered saline solution containing 2% sucrose at pH 7.0 at bioactive compound concentrations of 10, 30, 100, and 300 μM. The concentrations of the bioactive compound encapsulated within the nanotubes were recalculated by applying the encapsulation percentage obtained for each compound. A 10 mM buffer solution was used to avoid osmotic stress. *Triticum aestivum* L. cv. Burgos was selected. Logran was employed as a positive control in the bioassay.

### Phytotoxicity Bioassays

Three weed species, namely, the monocotyledons perennial ryegrass (*L. perenne* L.), the reed canary grass (*P. arundinacea* L.), and the dicotyledon common purslane (*P. oleracea* L.), were evaluated as target plants in this bioassay. *L. perenne* L. seeds were purchased from Herbiseed (Reading, UK). *P. oleracea* L. and *P. arundinacea* L. were purchased from Cantueso Natural Seeds (Córdoba, Spain). Bioassays were conducted using Petri dishes (50 mm diameter) with one sheet of Whatman no. 1 filter paper. Germination and growth were conducted in aqueous solutions at controlled pH using 10^–2^ M of 2-[*N*-morpholino]ethanesulfonic acid and 1 M NaOH (pH 6.0). The compounds to be assayed were dissolved in buffer, while nanotube solutions were just diluted, and test concentrations of 10^–3^, 3 × 10^–4^, 10^–4^, 3 × 10^–5^, and 10^–5^ M were prepared. In the case of nonencapsulated compounds, 0.05% v/v of dimethyl sulfoxide (DMSO) was applied as the solubility enhancer. Parallel controls were also run as described previously for coleoptile bioassays. Four replicates containing 20 seeds were used. Treatment, control, or internal reference solution (1 mL) was added to each Petri dish. After adding the seeds and aqueous solutions, the Petri dishes were sealed with Parafilm to ensure closed-system models. Seeds were further incubated at 25 °C in a Memmert ICE 700 controlled environment growth chamber. The photoperiod was 24 h of dark for all weeds. Bioassays took 6 days for perennial ryegrass, 8 days for reed canary grass, and 4 days for common purslane. After growth, plants were frozen at −10 °C for 24 h to avoid subsequent growth during the measurement process. The parameters evaluated (germination rate, root length, and shoot length) were recorded using a Fitomed system, which allowed automatic data acquisition and statistical analysis using the associated software. Data were analyzed statistically using Welch’s test, with significance fixed at 0.01 and 0.05. The results are presented as percentage differences with respect to the control. Zero represents control, positive values represent stimulation, and negative values represent inhibition.

## Results and Discussion

### Sesquiterpene Lactone Characterization

Compounds **1**, **2**, and **3** were isolated following the experimental procedure described in the experimental section. Although these compounds are obtained in only a very low percentage yield (1.2–3.9·10^–3^% as wt % of dry plant material), secondary metabolites such as these are not usually isolated on a gram-scale. This could allow the broad application of these compounds on a farm scale. Although enriched fractions were employed herein to obtain the compounds of interest, it may be possible to isolate larger quantities from other fractions that also contain these sesquiterpene lactones. Specifically, according to Rial et al.,^4^ aguerin B (**1**), cynaropicrin (**2**), and grosheimin (**3**) can be isolated in 0.48, 5.13, and 0.77% yield with respect to the starting extract, respectively.

Bioavailability is one of the most relevant values irrespective of whether crop or soil application is to be used. In terms of natural products that could be used as herbicides, the compromise between lipophilicity and water solubility is an important aspect that determines the subsequent effect on weeds. Thus, although previous studies published^3^ with these kinds of guaiane derivatives have used DMSO as the codissolvent to achieve their remarkable phytotoxicity results, this nongreen approach would be difficult to use in agriculture.

In order to find a green application of natural products on a larger scale, researchers in this field tend to follow a limited number of different approaches. Chemical derivatization of the bioactive products is perhaps one of the best knowns but can lead to side effects and a loss of natural properties, which in the case of secondary metabolites can result in nonpersistence and a low lifetime due to easy recognition and metabolization by other plants and microorganisms. According to Macías et al.,^16^ maintaining the natural product as unmodified as possible is the best approach to generate a new generation of herbicides. As such, encapsulation in a host for the guaiane-type bioactive compounds, which does not result in a covalent modification of the natural product, will avoid the need for any organic solvent.

Lithocholic acid (**7**), a natural steroid, is the host selected for encapsulation herein due to its ability to self-arrange into nanotubes. The colloidal properties of the resulting nanotube structure allow the transport of guest compounds.^17^ Moreover, the supramolecular structure exhibited by this compound is stable in the neutral pH range, thus meaning that problems related to size toxicity are overcome when the full structure enters a predominantly acidic cell medium. In addition, this is also a natural product, thus enhancing the green nature of the resulting agrochemical. Finally, this structure has been tested previously in phytotoxic bioassays, which demonstrated its innocuous nature.^12^

Going back to the physicochemical properties, Table 2 shows that **1** and **3** have a very low water solubility. The solubility of **1** is slightly lower than that of **3** as the unsaturated ester increases the lipophilicity, while the hydroxyl and ketone groups in **3** are more polar. Cynaropicrin (**2**) shows the highest water solubility, as expected due to the presence of two hydroxyl groups, although the value obtained cannot easily be explained.

**Table 2:** Maximum Concentration of Sesquiterpene Lactones That Can Be Dissolved in Water at 25 °C

compound	solubility at 25 °C (mg/L)	% RSD^a^
aguerin B (**1**)	148.2	4.7
cynaropicrin (**2**)	817.3	2.1
grosheimin (**3**)	106.4	2.5

a Relative standard deviation.

According to Table 2, the water solubility of these sesquiterpene lactone is higher than that of others, such as dehydrocostuslactone and costunolide, which have values of 5.1 and 26.0 mg/L, respectively. Nevertheless, these higher-solubility values appear to have little effect on phytotoxicity as the IC_50_ values for **1**, **2**, and **3** are very similar to those for dehydrocostuslactone and costunolide, despite the solubility values varying 20-fold.^18^

**Figure 2:**
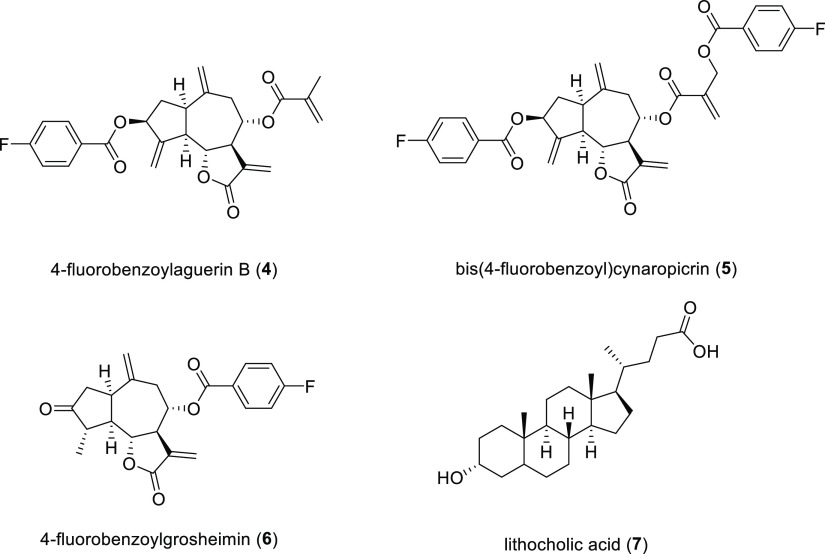
Fluorinated sesquiterpene lactone derivatives **4–6** employed in encapsulation studies, along with the structure of lithocholic acid (**7**), the supramolecular structure of which is the organic nanotube.

Encapsulation in lithocholic acid nanotubes will allow better transport of the sesquiterpene lactones to cells, thereby decreasing the amount of compound that needs to be applied to achieve phytotoxicity and avoiding the use of organic solvents. However, this is a complex process that is difficult to follow using standard spectroscopic methods due to the similar elemental composition of the host and guest (C, H, and O). As such, differentiating between correct encapsulation in a core/shell structure and adsorption on the surface of the organic material is a challenge. In order to ensure successful characterization, the approach proposed herein involves a quantitative reaction carried out to introduce a differentiating element, namely, a halogen atom. Thus, the 4-fluorobenzoyl esters of sesquiterpene lactones isolated from *C. cardunculus* were generated before encapsulation and the fluorine atom was monitored by TEM microscopy to discern the location of the molecule with regard to the organic nanotubes (Figure 2). These results with fluorinated compounds will be useful for understanding the behavior of natural compounds when they are encapsulated in similar structures.

### NMR Characterization

The portion of the ^13^C NMR spectrum for the fluorinated fragment shows multiplicity due to the NMR-active fluorine atom. The nuclear spin of ^19^F is known to generate doublets for neighboring carbon atoms, the most remarkable being the C-5 position of the aromatic ring, which exhibits a coupling constant of more than 250 Hz. Furthermore, the hydrogen atom alpha to the aromatic ester presents a high shift in comparison with natural sesquiterpene lactones. In the case of bis(4-fluorobenzoyl)cynaropicrin (**5**), H-3 and H-4′ are shifted from 4.55 to 5.76 and from 4.37 to 5.10 ppm, respectively. Oxidation of the hydroxyl group to form the ester also affects the beta-position, and the H-2 protons are also deshielded, with the same shift being observed for both derivatives at the C-3 position: 4-fluorobenzoylaguerin B (**4**) and bis(4-fluorobenzoyl)cynaropicrin (**5**).

With regard to the NMR data for the aromatic ring, when one fluorine atom is present as a substituent, the *ortho*-carbon appears as a doublet (*J* = 22.0–22.3 Hz), as do the *meta*-carbons, although with smaller coupling constants (*J* = 9.4–9.5 Hz). The carbon in the *para* position exhibits the smallest coupling constant of around 2.8–3.0 Hz. The ^1^H NMR spectra are also affected by the halogen, and the multiplicity becomes much more complex.

### Organic Nanotube Synthesis and Characterization

Once the derivatives had been obtained, encapsulation was carried out. Thus, lithocholic acid was dissolved in NaOH solution along with the fluorinated compound and neutralization was performed by the dropwise addition of HCl solution. This in situ encapsulation method was designed to increase the encapsulation percentage as nanotube formation, while these fluorobenzoyl derivatives present in the solution increase the possibility of the compound becoming confined during micellar aggregation, as reported in the literature.^12^

Purification by dialyzing the sample allowed the removal of NaCl crystallites generated as byproducts during nanotube formation. Furthermore, all the molecules that had not been trapped inside the nanotubes could be removed after dialysis with a 10,000 Da cutoff. The aqueous solutions employed in the synthesis were prepared using type-I water in order to decrease the presence of ions that could increase salt production. Following the method reported by Mejías et al.,^12^ dialysis was performed for 10 min to preserve the cohesiveness of the supramolecular structure.

**Figure 3:**
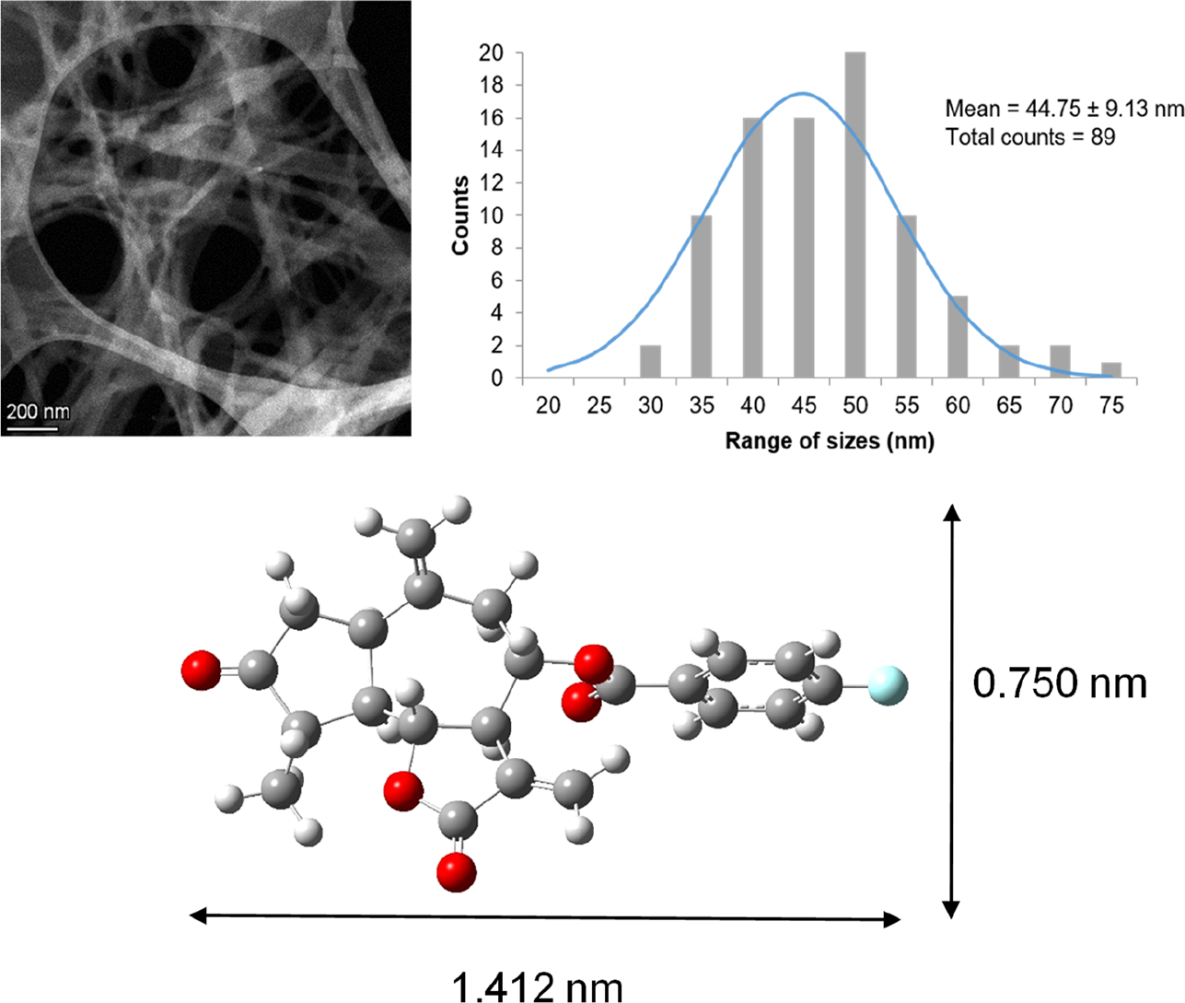
(Top) HAADF image and diameter size distribution of the nanotubes encapsulated with fluorinated derivatives. (Bottom) DFT-B3LYP/6-31G(d,p) calculation for the size of 4-fluorobenzoylgrosheimin

TEM characterization was carried out with 4-fluorobenzoylgrosheimin (**4**) as an example to provide chemical information about the spatial location of the fluorine-labeled encapsulated molecule and host. In particular, the nanotube size was measured directly from the HAADF low-magnification images, and a size histogram for 89 nanotubes (Figure 3) gave a mean size diameter of 44.75 ± 9.13 nm (Figure 3). This value is in the range of those obtained for empty nanotubes (49.79 nm) in the literature.^12^Figure 3 shows that the size of **4** is 1.412 × 0.750 nm, which is much lower than the internal diameter of the nanotube cavity; therefore, this physical confinement should not alter the size distribution of the supramolecular structure.

**Figure 4:**
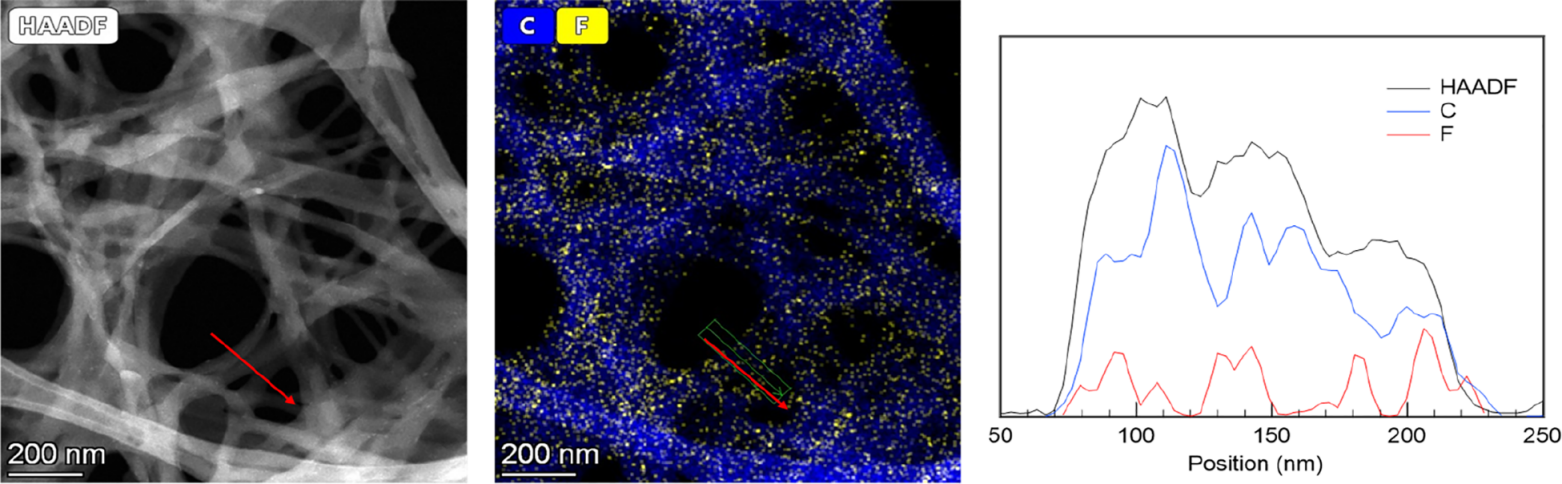
(Left) HAADF image of nanotubes. (Middle) STEM–XEDS maps of different elements present in the nanotubes hosting fluorinated derivatives. (Right) Elemental profile along the area analyzed in the maps.

Nanoanalytical studies confirmed that the fluorinated derivatives had indeed been encapsulated. Thus, the XEDS analysis of the nanotube@**4** sample (Figure 4) illustrated the spatial distribution of C (0.277 keV) and F (0.675 keV), with the C, F, and HAADF intensity profile extracted from the area marked with a red arrow showing that the C and F signals are anticorrelated. In particular, the F signal is observed when the C signal is reduced, as would be expected for the empty central part of the nanotube, thus indicating that the fluorinated derivatives are located inside the nanotube.

**Figure 5:**
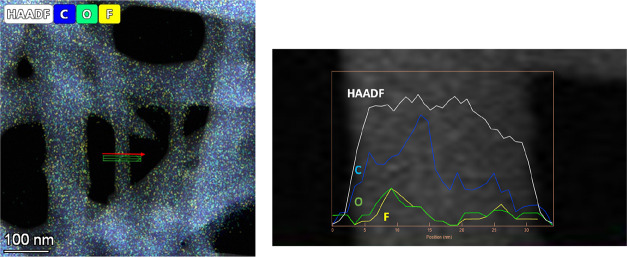
(Left) STEM–XEDS maps of C, O, and F present in the nanotubes hosting fluorinated derivatives. (Right) Elemental profile along the area analyzed in maps to correlate C, O, and F signals.

Apart from the fluorine signal, 4-fluorobenzoylgrosheimin (**4**) contains a higher number of oxygen atoms than lithocholic acid. As such, an increase in the oxygen signal in the cavity of the nanotube, together with the increase in the fluorine signal, must correlate with the presence of **4** inside the nanotube. Figure 5, in which the HAADF profile along nanotubes shows an increase in the oxygen signal whenever the fluorine signal increases, supports this hypothesis. Both signals arise when the carbon signal is reduced, thus indicating the start of the nanotube cavity and confirming the position of the guaiane-type lactone inside the nanostructure.

By applying an analogy-type approach, if the fluorinated derivatives of natural products fit in the nanotube cavity, sesquiterpene lactones must show the same behavior due to their structural similarity. As such, nanosystems were synthesized following the same methodology as for the fluorinated compounds and the resulting structures were also confirmed using electron microscopy techniques.

Encapsulation percentage is one of the most relevant values to be determined for these systems. To determine the quantity of the sesquiterpene lactones present inside the nanotubes, we disrupted the micellar structure using an organic solvent. Lithocholic acid is soluble in methanol, and the addition of this solvent disrupts the nanotube, thereby releasing the lactones. Table 3 shows that the encapsulation percentages obtained herein are smaller than those previously reported in the literature for herbicides. This could be due to the presence of the hydroxyl group, which may interfere with nanotube formation and stability. The main self-assembly mechanism accepted by the scientific community is hydrogen bond formation between the hydroxyl group of one lithocholic acid and the acid group of a neighboring molecule.^19^ Thus, the hydroxyl group in every sesquiterpene lactone could have interacted with the steroid acid, thereby decreasing the likelihood of entrapment inside the nanotube.

**Table 3:** Concentration of Sesquiterpene Lactones inside the Nanotubes

compound	concentration (mg/L)	% RSD	% encapsulation
aguerin B (**1**)	91.6	3.3	30.6
cynaropicrin (**2**)	119.1	5.7	40.1
grosheimin (**3**)	107.5	1.3	32.7

### Phytotoxic Bioassays

An etiolated wheat coleoptile bioassay was performed to evaluate whether the encapsulation procedure boosts the biological properties of the main *C. cardunculus* sesquiterpene lactones. This highly sensitive in vitro plant model bioassay provides information regarding general phytotoxicity.

**Figure 6:**
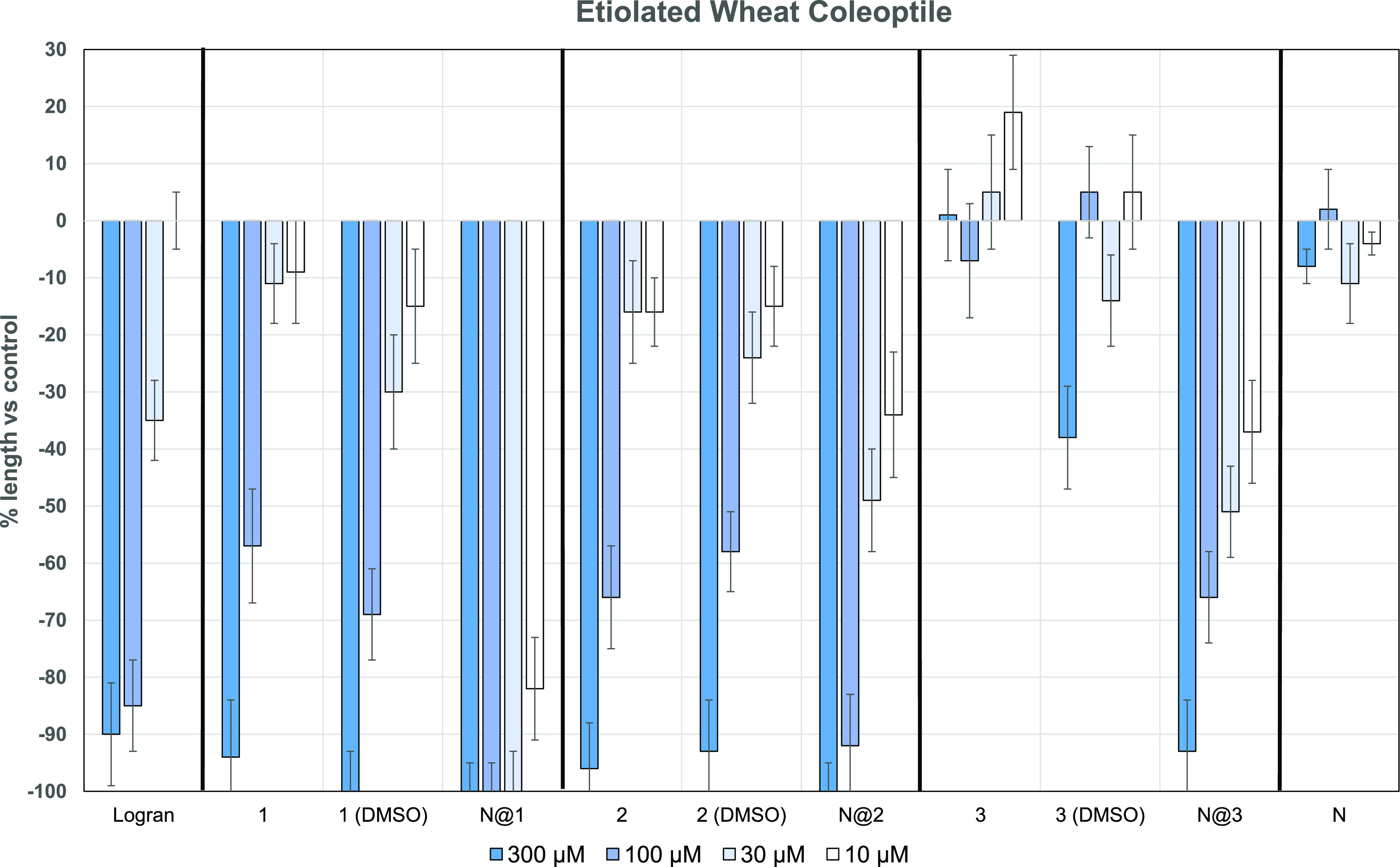
Etiolated wheat coleoptile bioassays for aguerin B (**1**), cynaropicrin (**2**), and grosheimin (**3**) in aqueous media. Same compounds applying an organic cosolvent (DMSO) [**1 (DMSO)**, **2 (DMSO)**, and **3 (DMSO)**] and encapsulated in nanotubes also in aqueous media (**N@1**, **N@2**, and **N@3**). A positive control (**Logran**) and empty nanotubes (**N**) were also tested.

Figure 6 shows that aguerin B (**1**) and cynaropicrin (**2**) exhibit remarkable inhibitory activity at 300 μM when tested in aqueous solution (at pH 7.0) and an organic solvent. The values obtained for both these substances are better than those obtained for the commercial herbicide Logran at the same concentration. However, at the next value tested (100 μM), the bioactivity decreased to almost half of that for the previous concentration, which is not observed for the commercial herbicide. Furthermore, the same profile was obtained at the concentrations of 30 and 10 μM. An inhibitory activity of 40% is obtained for grosheimin (**3**) when dissolved in DMSO, thus indicating that this substance cannot be used as an agrochemical under these conditions.

The values obtained for encapsulated sesquiterpene lactones (NTs) show much better growth inhibition when the compounds are encapsulated and dispersed in an aqueous medium compared with the free compound applied in an organic cosolvent (DMSO). The most remarkable case is aguerin B (**2**), which inhibits 100% of growth at 30 μM and more than 80% of growth at 10 μM. The results obtained for both **1** and **2** exceed the values observed for Logran. Although encapsulated grosheimin (**3**) shows an astonishing increase in inhibitory activity in comparison with its aqueous solution, the values obtained are still lower than those for the other compounds. Table 4 shows a comparison between the IC_50_ values for the different formulations. When a guaiane compound is encapsulated in the organic nanotube, the resulting IC_50_ value is better than that obtained when using an organic solvent or buffer. Furthermore, the NT values for **1**, **2**, and **3** are twice than those for the commercial herbicide Logran (IC_50_ = 42.53). It was not possible to calculate the IC_50_ value for encapsulated aguerin B (**1**) as lower concentrations cannot be reached in the bioassay without compromising the nanostructure. All these values correspond to the boosted properties of the sesquiterpene lactones as empty nanotubes have no effect on wheat coleoptiles.

**Table 4:** IC_50_ Values for Different Formulations of the Main Sesquiterpene Lactones from *Cynara cardunculus*^a^

compound	**1** (buffer)	**1** (DMSO)	**1** (NTs)	**2** (buffer)	**2** (DMSO)	**2** (NTs)	**3** (Buffer)	**3** (DMSO)	**3** (NTs)
IC_50_ (μM)	84.75	54.36	<10	69.78	73.24	24.79	>300	>300	28.80
S.D (%)	10.59	6.99		10.36	6.47	5.93			8.24
*R*^2^	0.9609	0.9796		0.9592	0.9827	0.9788			0.9670

a Statistical analysis with *p* < 0.05.

Upon comparing the modulation of the physicochemical properties of these guaiane compounds with the bioassay results, it seems clear that water solubility is insufficient to explain the growth inhibition of the wheat coleoptile. Table 3 shows that the encapsulation percentage does not reach the water solubility limit; therefore, this must not be the most relevant aspect affecting the bioactivity. In this regard, the transport property, which is directly related to bioavailability, could be a more significant contribution as a result of the natural product used to form the nanotube. We believe that lithocholic acid may be easily recognized by exomembrane proteins and, together with the solubility and side-reaction prevention of the encapsulated compound, may allow the target to be reached better than when an organic solvent is used as the vehicle.

**Figure 7:**
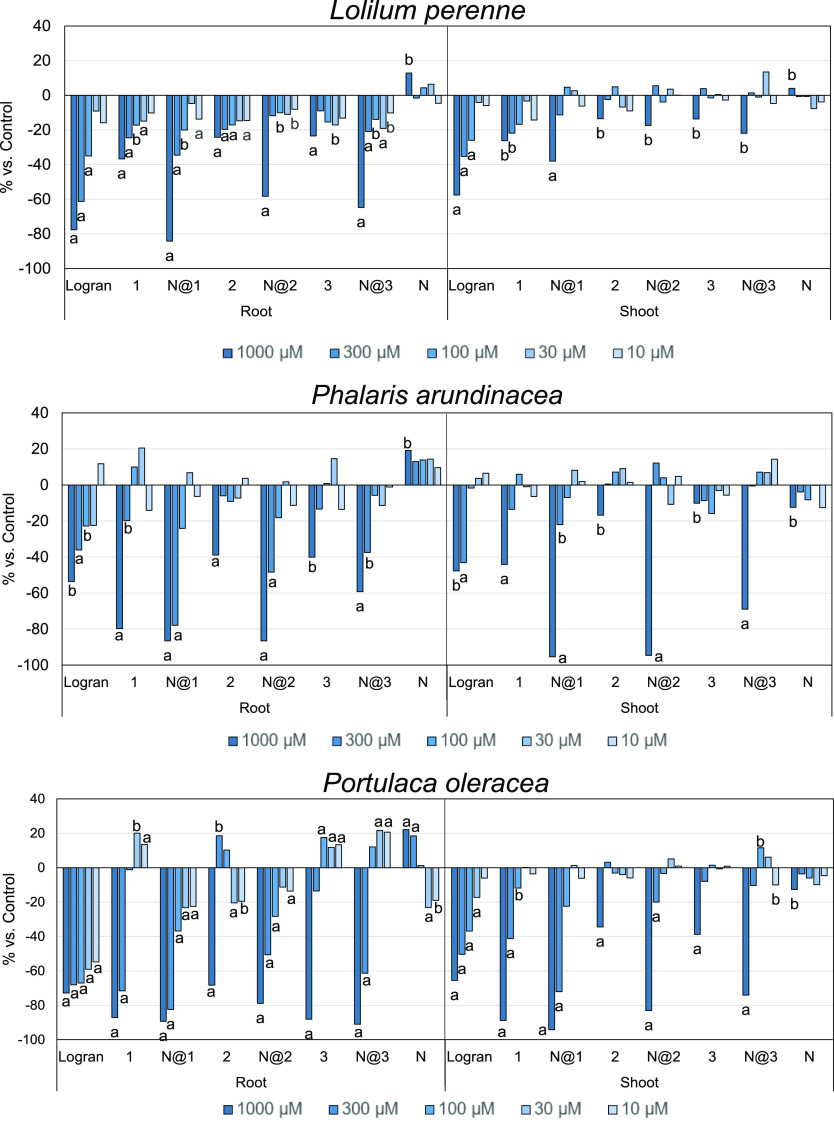
Effects of aguerin B (**1**), nanotube@aguerin B (**N@1**), cynaropicrin (**2**), nanotube@cynaropicrin (**N@2**), grosheimin (**3**), nanotube@grosheimin (**N@3**), and nanotube (**N**) on roots and shoots of relevant weeds. Data were analyzed statistically using Welch’s test, with significance fixed at 0.01 (a) and 0.05 (b).

Phytotoxicity bioassays for weeds that typically infect the main cereal and other important crops, especially in North America,^6,7,13^ were performed to determine how nanotube encapsulation affects the main parameters. Germination, root and shoot length, and geometry were analyzed in the in vitro bioassay for *P. arundinacea*, *L. perenne* L., and *P. oleracea* L. In common with the wheat coleoptile bioassay, empty nanotubes were found to be innocuous to weeds; therefore, the phytotoxicity displayed by the samples is only due to the molecules encapsulated therein. For every weed, compounds **1**, **2**, and **3** were tested in free and encapsulated form and, as shown in Figure 7, all nanotube-encapsulated formulations were found to improve the results for the free form. In the case of *L. perenne*, the first concentrations of encapsulated compounds present high inhibition activity (**N@1**, **N@2**, and **N@3**), whereas subsequent concentrations show a quick reduction in it. In addition, the activity shown by **N@1** against roots, shoots, and germination (Figure S1) exceeds those for both the free compound and the positive control (Logran).

The results for *P. arundinacea* are similar, with encapsulated sesquiterpene lactones showing higher inhibition than their free counterparts. Every activity achieved with the 1000 μM encapsulated form doubled the value achieved for the positive control. In addition, in the case of roots, even the second concentration tested exceeded the values obtained for Logran. This trend is similar to that observed with *L. perenne* and *P. oleracea*, thus highlighting the effect of sesquiterpene lactones on the mechanism regulating root growth.^20^ However, the values for shoots show a marked reduction in inhibition after the dilution process. The germination values observed for *P. arundinacea* (Figure S1) are statistically significantly different in the case of **N@1**, **N@2**, and **N@3**, as are those for *P. oleracea*. Furthermore, germination of this latter weed is not inhibited by Logran, whereas nanotube encapsulation of *C. cardunculus* sesquiterpenes allows this parameter to be controlled.

The roots and shoots of *P. oleracea* are the most sensitive. Thus, all encapsulated lactones present inhibition values of almost 100% for root length when the compounds are encapsulated and more than 60% at the second concentration tested (300 μM). In the case of the free compound, the activities are always lower than those for the nanotube-encapsulated compounds. Furthermore, a similar trend is observed for shoots, with encapsulation enhancing the already active sesquiterpenes.

In conclusion, we have generated a new kind of herbicide formulation by using the supramolecular structure of a natural product to encapsulate different natural guiaiane-type sesquiterpene lactones. The compounds isolated from *C. cardunculus* have been modified to introduce a fluorine atom in the main structure, thus allowing us to determine their position with respect to the nanotube, in order to confirm encapsulation, using STEM techniques. XEDS analysis also confirmed the correct encapsulation upon determining the oxygen and fluorine profile across the nanotube section, thus showing that the natural product derivative is hosted in the nanotube cavity.

After confirming the presence of the compounds inside the nanotube, the same procedure was applied to the natural sesquiterpene lactones **1**, **2**, and **3**. These compounds showed a mean encapsulation percentage of around 30%, which was sufficient to exceed the phytotoxic values obtained upon dissolution of the free compounds in organic media. According to the IC_50_ values obtained from an *in vitro* wheat coleoptile assay, we have generated a nanoformulation method with twice the phytotoxicity of the commercial herbicide Logran. Furthermore, application of the nanotube-encapsulated formulations to weeds that commonly infect most cereal and other relevant crops, such as wheat and carrots, with relevance all over the world, and specifically in North America, reinforces the use of this vector to expand allelopathy. Germination and root and shoot inhibition assays for *P. arundinacea*, *L. perenne,* and *P. oleracea* showed that the encapsulated *C. cardunculus* sesquiterpene lactones exhibit higher activity than the free compounds. Furthermore, they exceed the values obtained for current herbicides. This will allow the application of very small quantities of the natural product as a new and green agrochemical with a low environmental impact.

## Supporting Information Available

^1^H NMR spectra, ^1^H NMR data of all compounds, and additional graphics of weed bioassay (PDF)
