# Supplementary material for: Encapsulation of Cynara Cardunculus Guaiane-type Lactones in Fully Organic Nanotubes Enhances Their Phytotoxic Properties
Source: J Agric Food Chem. 2022 Mar 15;70(12):3644–53. doi: 10.1021/acs.jafc.1c07806 (PMC8972271; doi:10.1021/acs.jafc.1c07806)
Supplement: Supplementary file 1 — jf1c07806_si_001.pdf [file jf1c07806_si_001.pdf]

Supporting Information

Encapsulation of *Cynara Cardunculus* Guaiane-type Lactones in Fully Organic Nanotubes Enhances Their Phytotoxic Properties

*Francisco J.R. Mejías[†], Inmaculada P. Fernández[ǂ], Carlos Rial[†], Rosa M. Varela[†], José M.G. Molinillo[†], José J. Calvino[ǂ], Susana Trasobares[ǂ], Francisco A. Macías[†]**

†Allelopathy Group, Department of Organic Chemistry, Institute of Biomolecules (INBIO), Campus CEIA3, School of Science, University of Cádiz, C/ República Saharaui, 7, 11510-Puerto Real (Cádiz), Spain

ǂDepartamento de Ciencia de los Materiales e Ingeniería Metalúrgica y Química Inorgánica, Facultad de Ciencias, Universidad de Cádiz, C/ República Saharaui, 7, 11510-Puerto Real (Cádiz), Spain

*Corresponding author: famacias@uca.es

Table of Content

^{1}H-NMR spectra of aguerin B (**1**) S-2

^{1}H-NMR spectra of cynaropicrin (**2**) S-3

^{1}H-NMR spectra of grosheimin (**3**) S-4

NMR data of 4-fluorobenzoylaguerin B (**4**) S-5

^{1}H-NMR spectra of 4-fluorobenzoylaguerin B (**4**) S-6

NMR data of bis(4-fluorobenzoyl)cynaropicrin (**5**) S-7

^{1}H-NMR spectra of bis(4-fluorobenzoyl)cynaropicrin (**5**) S-8

NMR data of 4-fluorobenzoylgrosheimin (**6**) S-9

^{1}H-NMR spectra of 4-fluorobenzoylgrosheimin (**6**) S-10

Figure S1 S-11

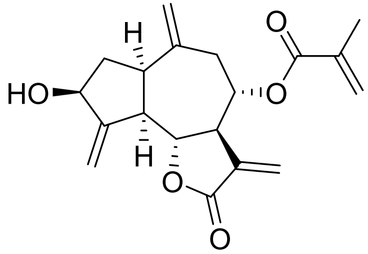
HO
H
H
O
O
O
O

Aguerin (**1**)

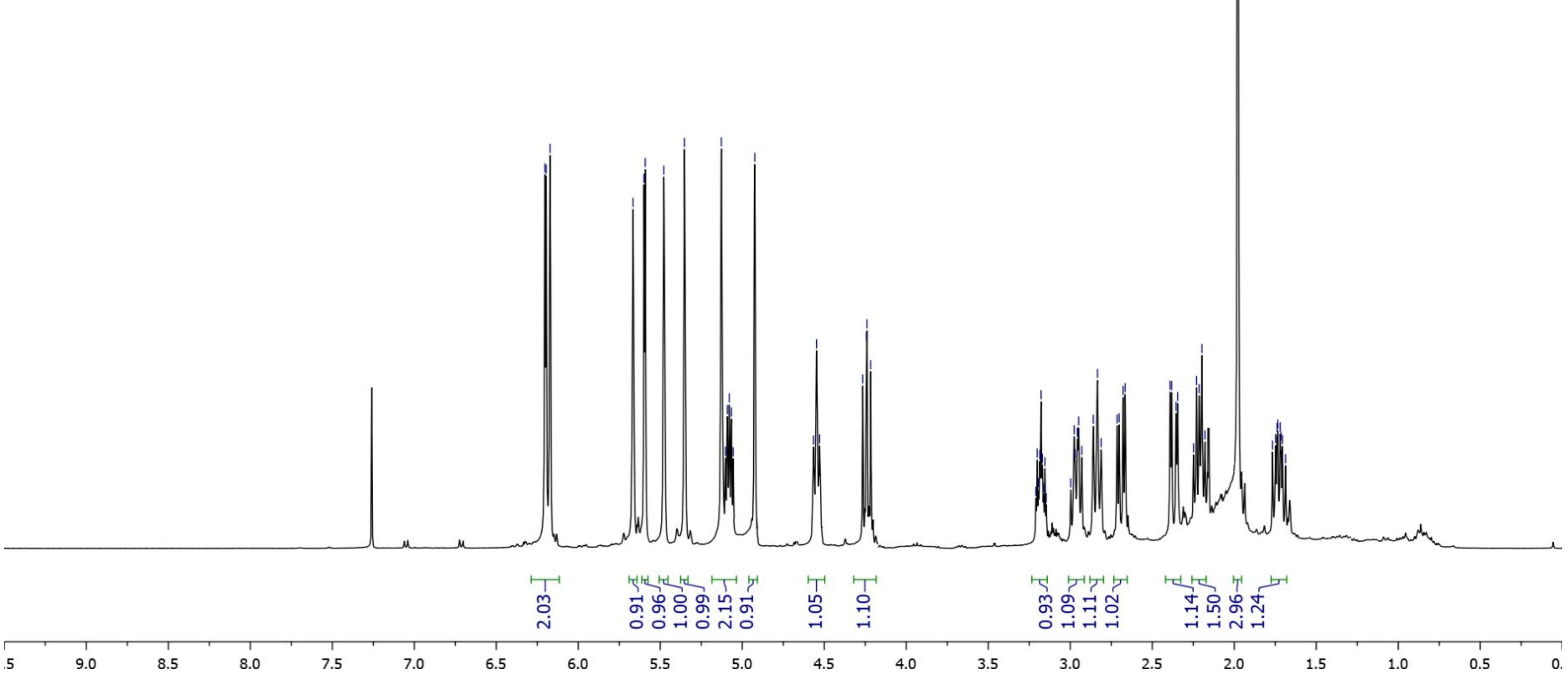
6.20 6.20 6.17 5.67 5.60 5.59 5.48 5.35 5.13 5.10 5.09 5.08 5.07 5.06 4.92 4.57 4.55 4.53 4.27 4.24 4.24 4.22 3.20 3.18 2.98 2.97 2.96 2.95 2.93 2.86 2.83 2.81 2.71 2.70 2.68 2.67 2.39 2.38 2.35 2.35 2.25 2.23 2.21 2.20 2.18 1.98 1.77 1.75 1.74 1.73 1.72 1.72 1.71
2.03 0.91 0.96 1.00 0.99 2.15 0.91 1.05 1.10 0.93 1.09 1.11 1.02 1.14 1.50 2.96 1.24
.5 9.0 8.5 8.0 7.5 7.0 6.5 6.0 5.5 5.0 4.5 4.0 3.5 3.0 2.5 2.0 1.5 1.0 0.5 0.

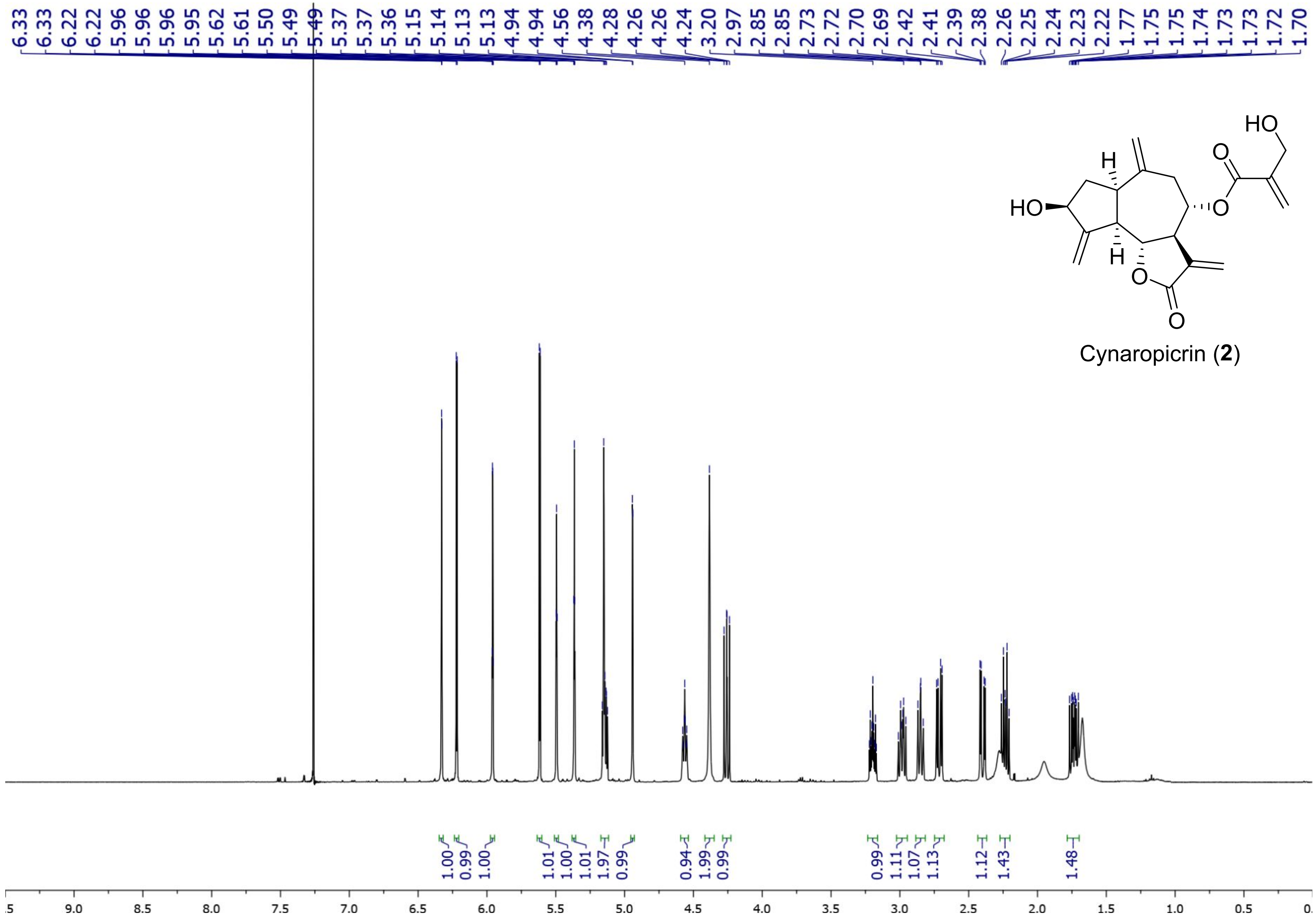
6.33 6.33 6.22 6.22 5.96 5.96 5.96 5.95 5.62 5.61 5.50 5.49 5.49 5.37 5.37 5.36 5.15 5.14 5.13 5.13 4.94 4.94 4.56 4.38 4.28 4.26 4.26 4.24 3.20 2.97 2.85 2.85 2.73 2.72 2.70 2.69 2.42 2.41 2.39 2.38 2.26 2.25 2.24 2.23 2.22 1.77 1.75 1.75 1.74 1.73 1.73 1.72 1.70
HO
O
H
HO
O
H
O
O
Cynaropicrin (2)
1.00 0.99 1.00 1.01 1.00 1.01 1.97 0.99 0.94 1.99 0.99 0.99 1.11 1.07 1.13 1.12 1.43 1.48
.5 9.0 8.5 8.0 7.5 7.0 6.5 6.0 5.5 5.0 4.5 4.0 3.5 3.0 2.5 2.0 1.5 1.0 0.5 0.

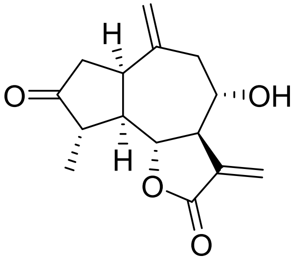

Grosheimin (**3**)

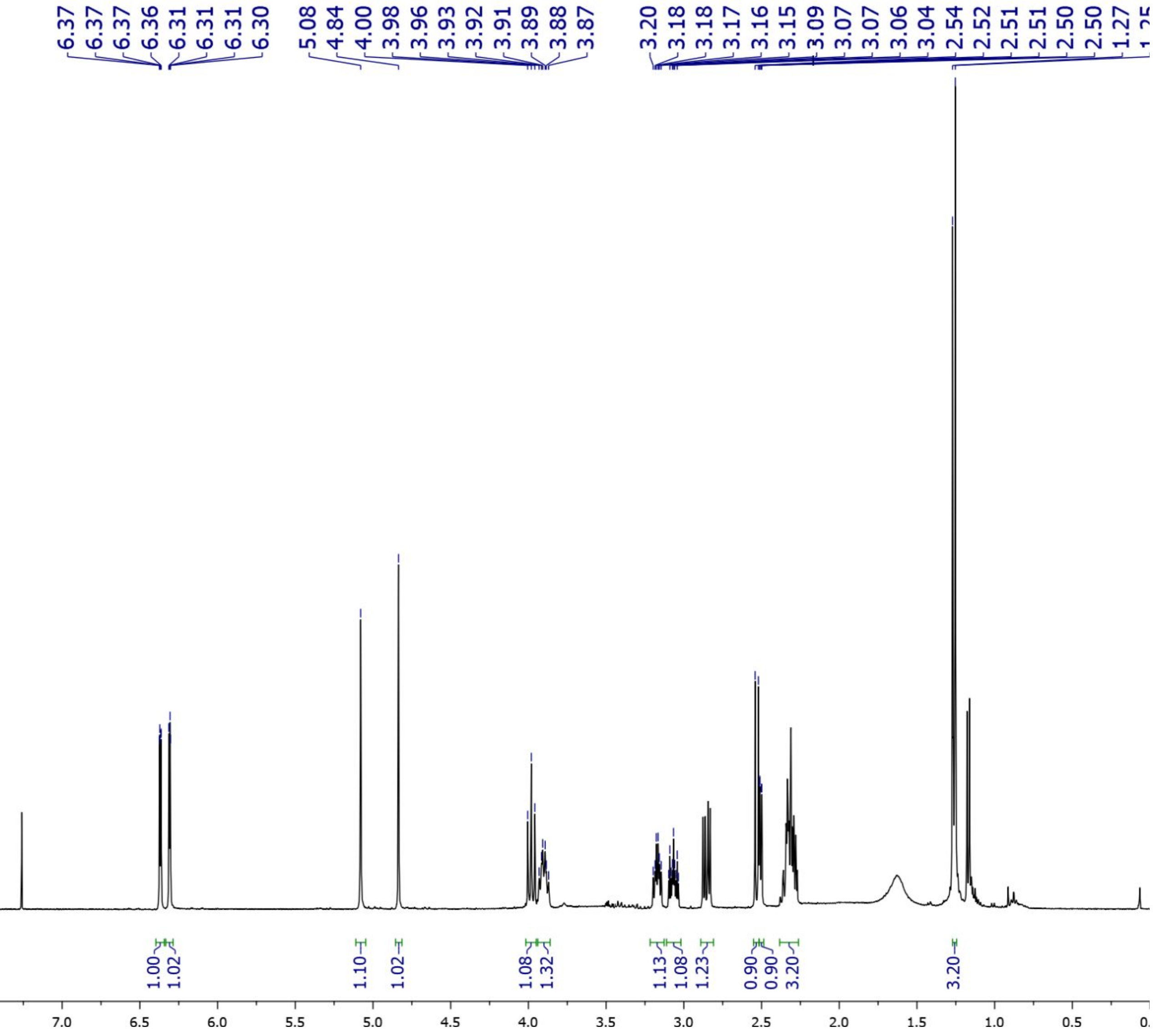

4-fluorobenzoylaguerin B (**4**)

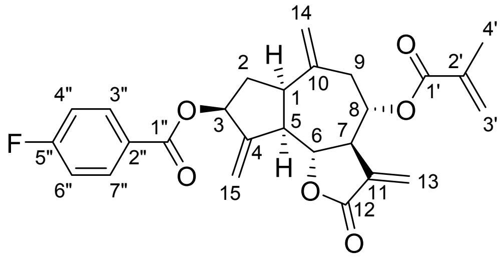

Atom	^{1}H	^{13}C
1	3.10 (ddd, J=10.8, 7.6, 7.6 Hz, 1H)	45.5
2	2.50 (ddd, J=13.7, 7.6, 7.6 Hz, 1H)<br>1.92 (ddd, J=13.7, 10.8, 7.4 Hz, 1H)	36.4
3	5.76 (dddd, J=7.5, 7.5, 1.9, 1.9 Hz, 1H)	75.4
4	-	147.0
5	2.93 (brdd, J=10.5, 9.0 Hz, 1H)	51.4
6	4.23 (dd, J=10.5, 9.1 Hz, 1H)	78.1
7	3.24 (dddd, J=9.5, 9.5, 3.2, 3.2 Hz, 1H)	47.7
8	5.11 (ddd, J=9.5, 4.8, 4.8 Hz, 1H)	74.0
9	2.72 (dd, J=14.5, 5.3 Hz, 1H)<br>2.40 (dd, J=14.5, 4.4 Hz, 1H)	37.5
10	-	141.3
11	-	137.2
12	-	169.0
13	6.25 (d, J=3.4 Hz, 1H)<br>5.64 (d, J=3.0 Hz, 1H)	122.9
14	5.17 (s, 1H)<br>4.99 (s, 1H)	118.4
15	5.60 (dd, J=1.8, 1.8 Hz, 1H)<br>5.44 (dd, J=1.8, 1.8 Hz, 1H)	116.2
1’	-	166.3
2’	-	135.9
3’	6.19 (brs, 1H)<br>5.69 (dd, J=1.5, 1.5 Hz, 1H)	126.7
4’	1.99 (brd, J=0.5 Hz, 3H)	18.2
1”	-	165.3
2”	-	126.2 (d, J=2.8 Hz)
3”, 7”	7.12 (dd, J=8.6, 8.6 Hz, 1H)	115.6 (d, J=22.0 Hz)
4”, 6”	8.06 (dd, J=8.6, 5.4 Hz, 1H)	132.2 (d, J=9.4 Hz)
5”	-	165.9 (d, J=254.1 Hz)

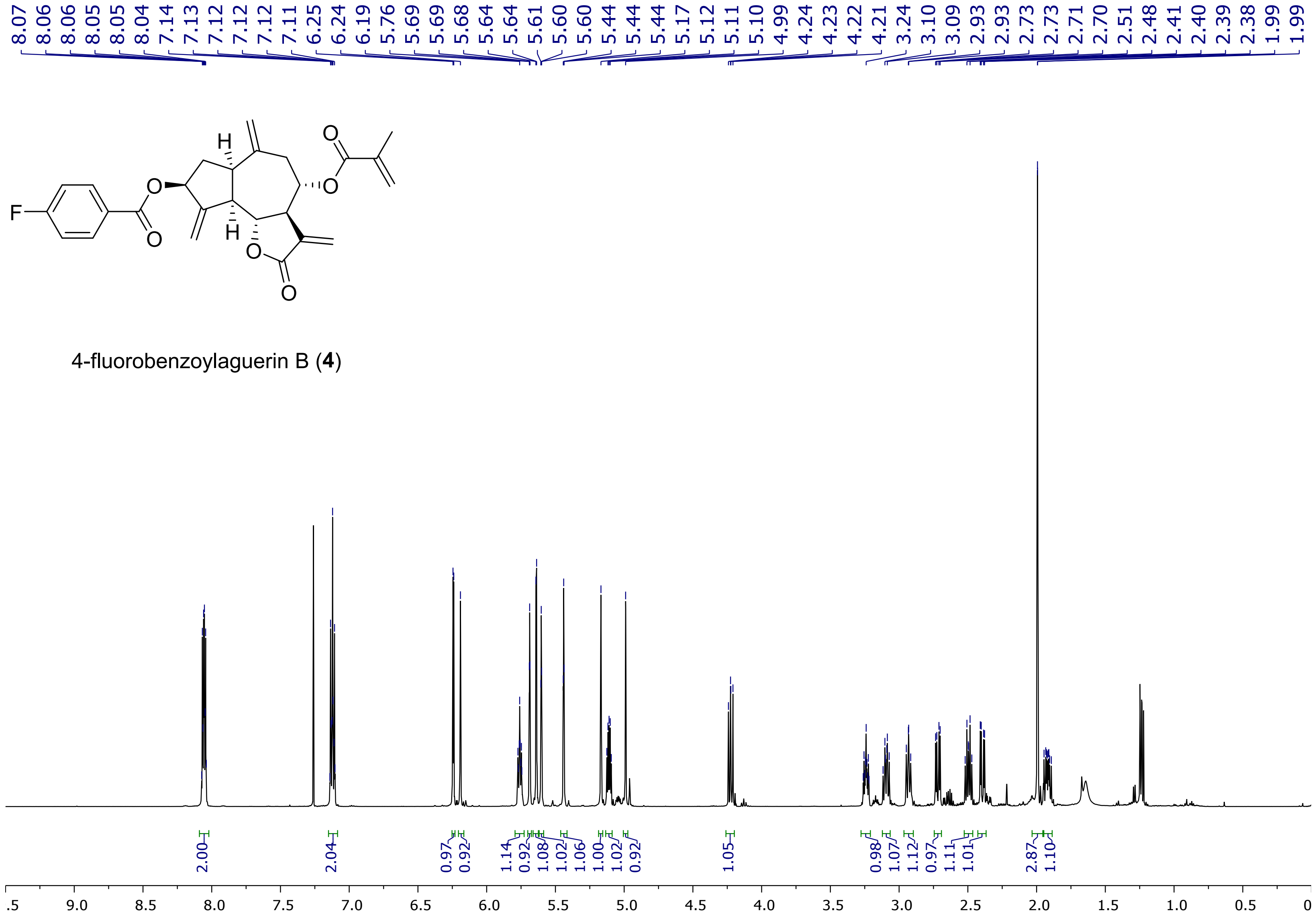

4-fluorobenzoylaguerin B (**4**)

bis(4-fluorobenzoyl)cynaropicrin (**5**)

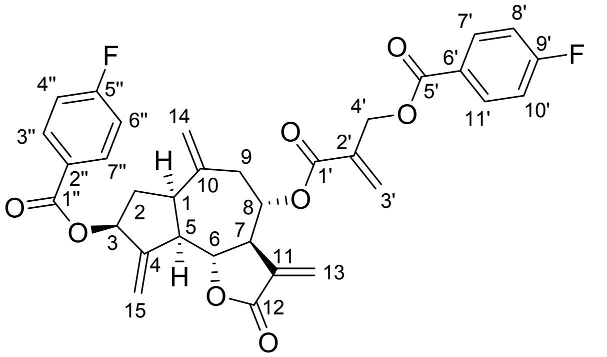

Atom	^{1}H	^{13}C
1	3.09 (ddd, J=7.6, 9.9, 9.9 Hz, 1H)	45.5
2	2.50 (ddd, J=7.6, 7.6, 14.4 Hz, 1H)<br>1.92 (ddd, J=7.6, 9.9, 14.4 Hz, 1H)	36.5
3	5.76 (dd, J=7.6, 7.6 Hz, 1H)	75.4
4	-	146.9
5	2.93 (dd, J=9.9, 9.9 Hz, 1H)	51.4
6	4.23 (dd, J=9.9, 9.9 Hz, 1H)	78.0
7	3.24 (dddd, J=3.2, 3.2, 9.2, 9.9 Hz, 1H)	47.7
8	5.19 (ddd, J=4.6, 4.6, 9.2 Hz, 1H)	74.5
9	2.73 (dd, J= 4.6, 14.5 Hz, 1H)<br>2.43 (dd, J= 4.6, 14.5 Hz, 1H)	37.4
10	-	141.1
11	-	137.1
12	-	168.9
13	6.21 (d, J=3.5 Hz, 1H)<br>5.63 (d, J=3.5 Hz, 1H)	122.8
14	4.98 (brs, 1H)<br>5.16 (brs, 1H)	118.7
15	5.45 (brs, 1H)<br>5.61 (brs, 1H)	116.4
1’	-	164.3
2’	-	135.1
3’	6.06 (brs, 1H)<br>6.51 (brs, 1H)	129.2
4’	5.10 (brs, 2H)	62.7
5’	-	167.1
6’	-	125.8 (d, J=3.0 Hz)
7’, 11’	8.05 (dd, J=5.7, 8.5 Hz, 2H)	132.21 (d, J=9.2 Hz)
8’, 10’	7.14 (dd, J=8.5, 8.5 Hz, 2H)	115.75 (d, J=22.2 Hz)
9’	-	166.0 (d, J=255.0 Hz)
1’’	-	165.4
2’’	-	126.2 (d, J=2.9 Hz)
3’’, 7’’	8.06 (dd, J=5.7, 8.5 Hz, 2H)	132.22 (d, J=9.4 Hz)
4’’, 6’’	7.14 (dd, J=8.5, 8.5 Hz, 2H)	115.73 (d, J=22.3 Hz)
5’’	-	165.9 (d, J=254.5 Hz)

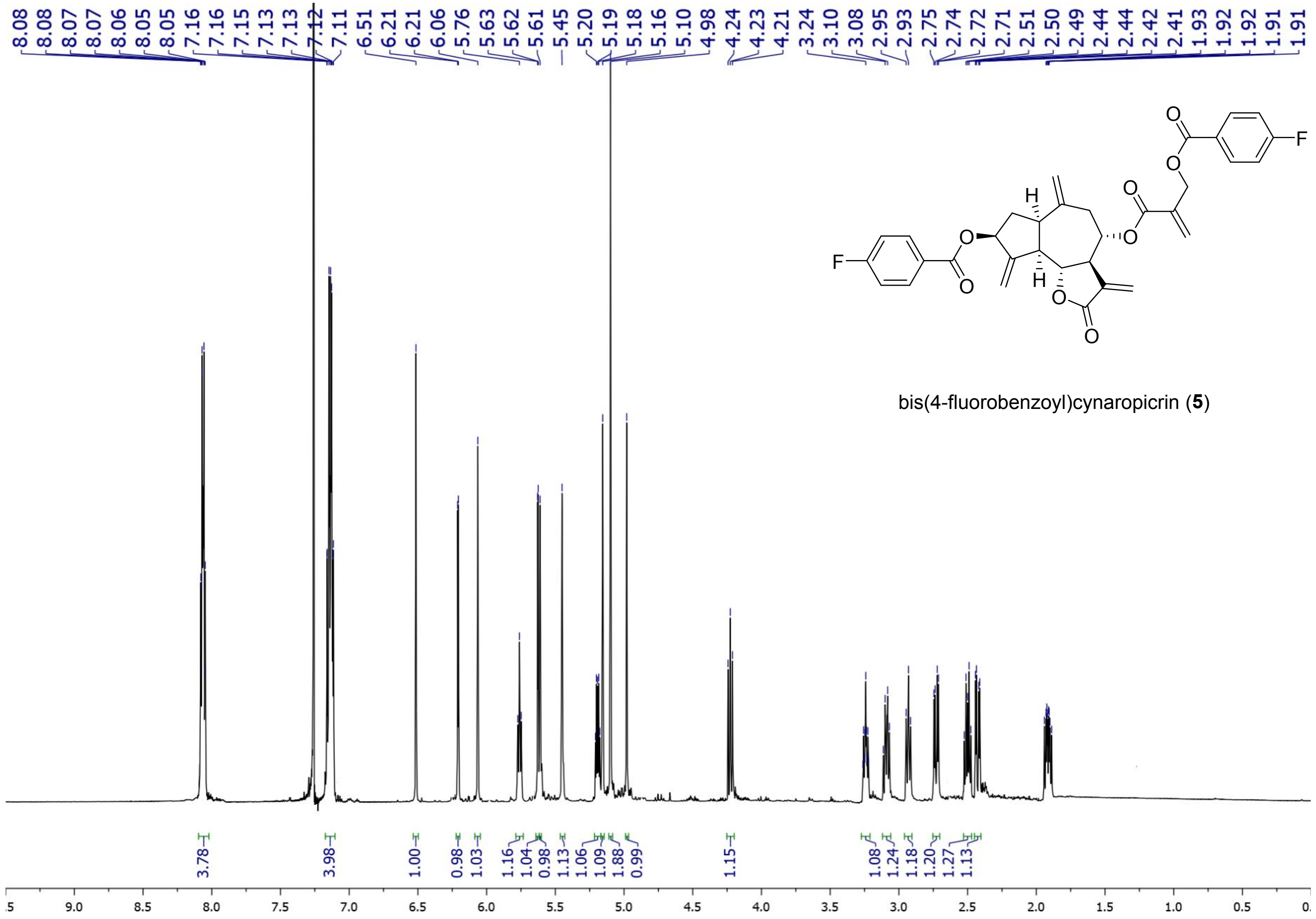

bis(4-fluorobenzoyl)cynaropicrin (**5**)

4-fluorobenzoylgrosheimin (**6**)

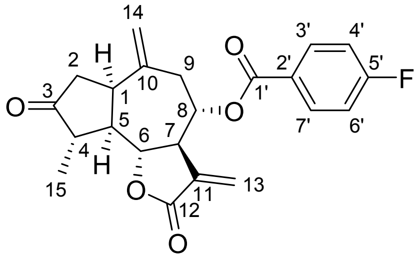

Atom	^{1}H	^{13}C
1	3.22 (ddd, J=12.8, 8.1, 4.8 Hz, 1H)	40.2
2	2.57 (dd, J=19.1, 8.5 Hz, 1H)<br>2.51 (ddd, J=19.1, 4.8, 1.0 Hz, 1H)	43.0
3	-	218.3
4	2.38 (m, 1H)[a]	46.9
5	2.37 (m, 1H)[a]	51.2
6	4.14 (dd, J=9.3, 9.3 Hz, 1H)	82.0
7	3.47 (dddd, J=10.5, 8.8, 3.2, 3.2 Hz, 1H)	46.7
8	5.24 (ddd, J=10.5, 5.9, 8.6 Hz, 1H)	74.8
9	2.38 (m, 1H)[a]<br>3.06 (dd, J=13.1, 5.9 Hz, 1H)	43.1
10	-	142.1
11	-	135.8
12	-	169.0
13	6.27 (d, J=3.3 Hz, 1H)<br>5.76 (d, J= 2.9 Hz, 1H)	124.9
14	5.18 (s, 1H)<br>4.91 (s, 1H)	117.0
15	1.28 (d, J=6.9 Hz, 3H)	14.9
1’	-	164.3
2’	-	125.5 (d, J=3.0 Hz)
3’, 7’	8.10 (dd, J=5.4, 8.9 Hz, 2H)	132.5 (d, J=9.5 Hz)
4’, 6’	7.17 (dd, J=8.9, 8.9 Hz, 2H)	116.0 (d, J=22.0 Hz)
5’	-	166.2 (d, J=255.2 Hz)

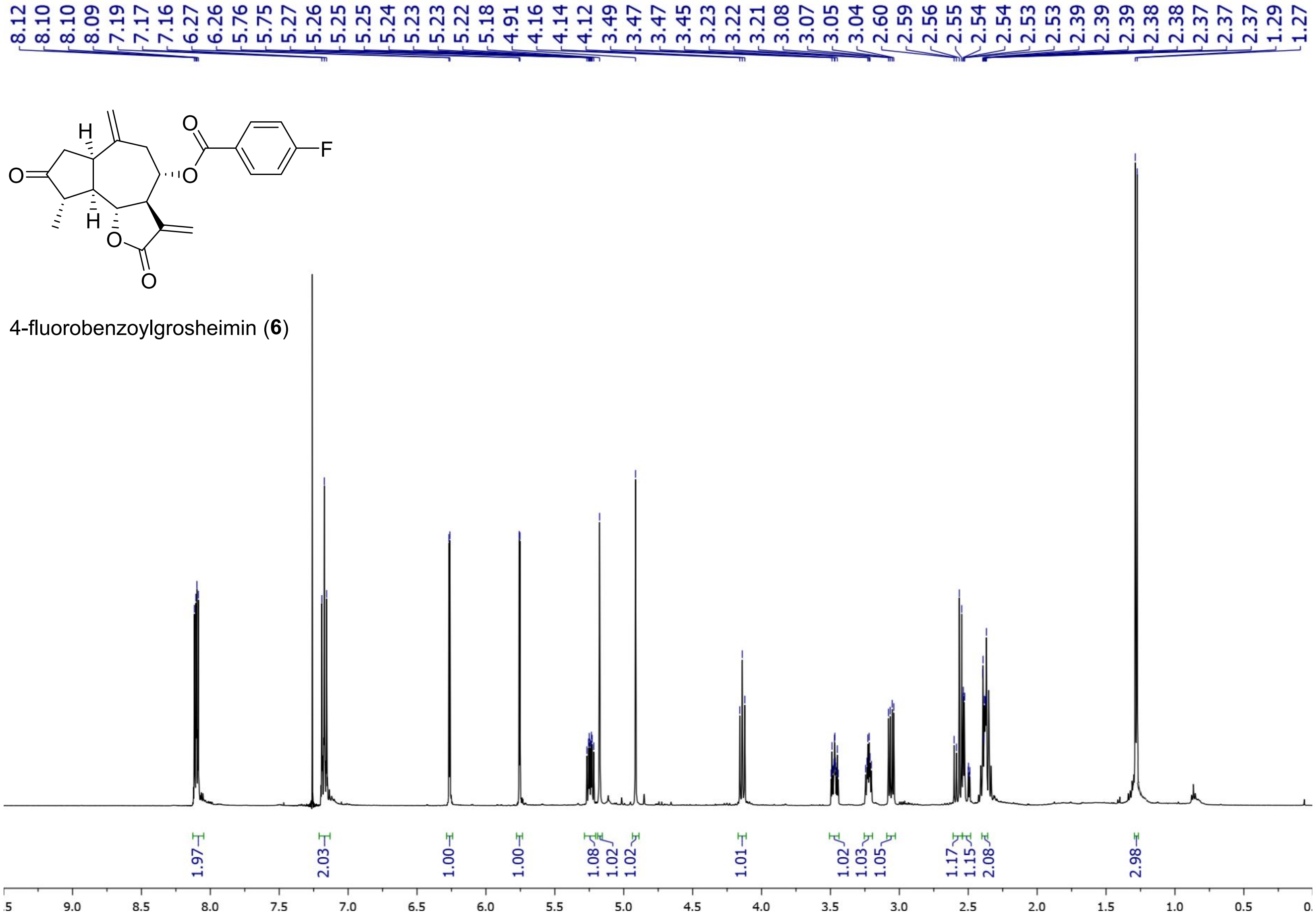

4-fluorobenzoylgrosheimin (**6**)

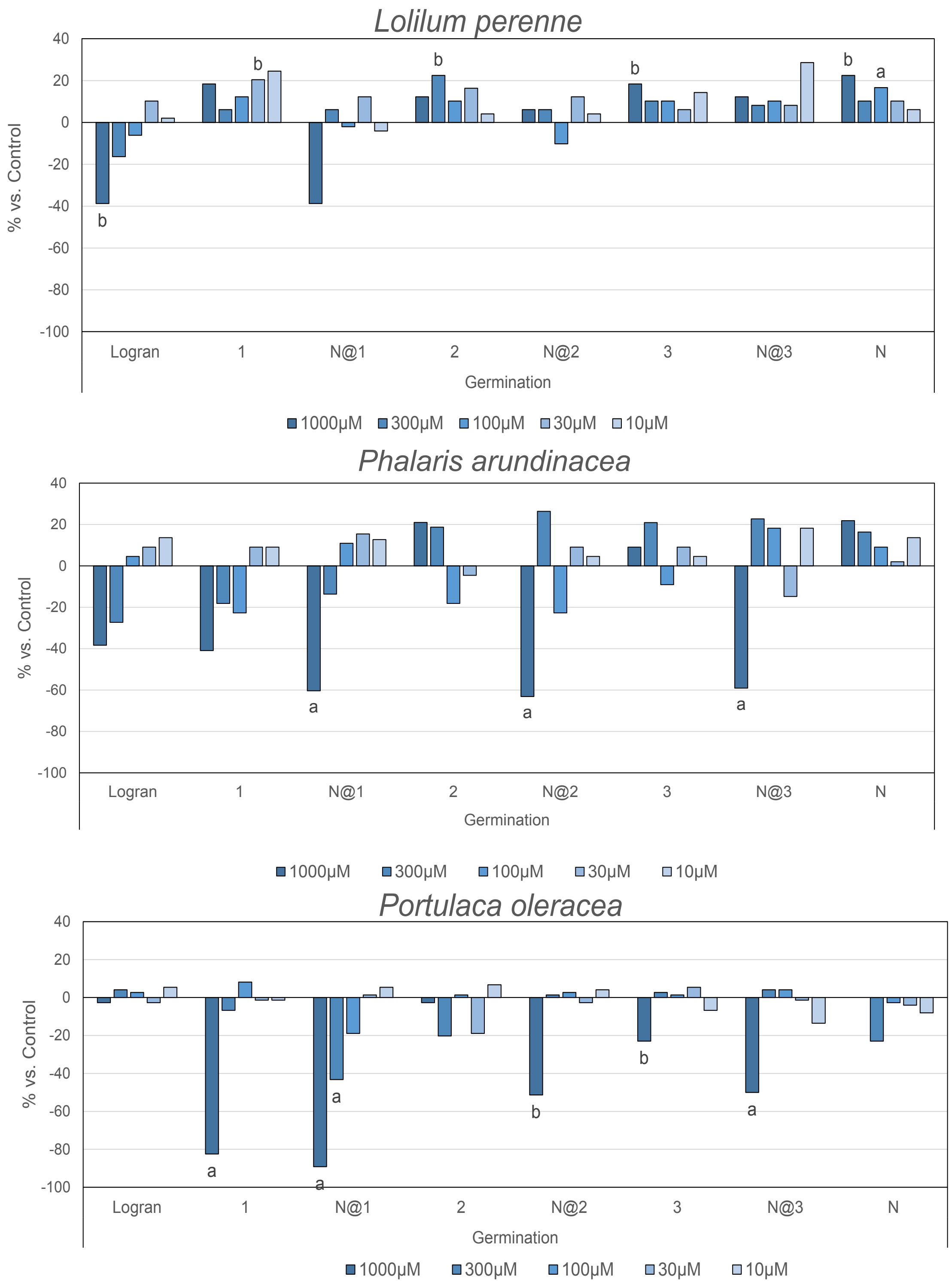

Figure S1. Effects of compounds aguerin B (**1**), nanotube@aguerin B (**N@1**), cynaropicrin (**2**), nanotube@cynaropicrin (**N@2**), grosheimin (**3**), nanotube@grosheimin (**N@3**) and nanotube (**N**) on germination of relevant weeds. Data were analyzed statistically using Welch's test, with significance fixed at 0.01 (a) and 0.05 (b).
